# The association of depressive symptoms with handgrip strength and gait speed in community-dwelling older adults: data from the baseline phase of Birjand Longitudinal Aging Study

**DOI:** 10.1186/s12877-024-04944-z

**Published:** 2024-05-03

**Authors:** Tina Nazari, Mitra Moodi, Hossein Fakhrzadeh, Huriye Khodabakhshi, Masoumeh Khorashadizadeh, Seyed Masoud Arzaghi, Shervan Shoaee, Mehdi Varmaghani, Hanieh-Sadat Ejtahed, Farshad Sharifi

**Affiliations:** 1 Department of Medical Geriatrics, School of Medicine, Tehran University of Medical Sciences, Tehran, Iran; 2 Social Determinants of Health Research Center, Birjand University of Medical Sciences, Birjand, Iran; 3 School of Health, Birjand University of Medical Sciences, Birjand, Iran; 4 Elderly Health Research Center, Endocrinology and Metabolism Population Sciences Institute, Tehran University of Medical Sciences, Tehran, Iran; 5 Social Determinants of Health Research Center, Mashhad University of Medical Sciences, Mashhad, Iran; 6 Obesity and Eating Habits Research Center, Endocrinology and Metabolism Clinical Sciences Institute, Tehran University of Medical Sciences, Tehran, Iran; 7 Endocrinology and Metabolism Research Center, Endocrinology and Metabolism Clinical Sciences Institute, Tehran University of Medical Sciences, Tehran, Iran

**Keywords:** Depression, Handgrip strength, Gait speed, Physical function, Older adults

## Abstract

**Background:**

Depression is a multifaceted condition with a high prevalence and burden to society. Handgrip strength (HGS) and gait speed (GS) are indices of physical health, which is linked to mental health. Previous studies have shown heterogeneity among countries in the association of physical parameters and depression. In this study, we aimed to investigate the association of HGS and GS with depressive symptoms in older adults.

**Methods:**

This is a cross-sectional study analyzing data from the Birjand Longitudinal Aging Study, a cohort of community-dwelling older adults (≥ 60 years old). Depressive symptoms were assessed by the nine-item Patient Health Questionnaire. HGS was measured with a hand dynamometer in a sitting position, and GS was estimated by a 15-foot walk test at usual pace.

**Results:**

Compared to participants in the first quartile, those in the second quartile of HGS had significantly lower odds of suffering from depressive symptoms, while GS was not significantly associated with depressive symptoms. A higher HGS was associated with a lower risk of moderate depressive symptoms, while a higher GS was related to a lower risk of moderately severe and severe symptoms.

**Conclusions:**

Our findings suggest that older people residing in Birjand, Iran with a moderate HGS are less likely to suffer from depressive symptoms than those with lower HGS.

**Supplementary Information:**

The online version contains supplementary material available at 10.1186/s12877-024-04944-z.

## Background

Depressive disorders, including major depressive disorder (MDD), depressive episodes, and dysthymia, are the most common and disabling mental disorders, with the highest prevalence among older adults [1]. These disorders are estimated to have a global prevalence of 31.74% in the elderly population [2]. Showing a 61.1% increase in disability-adjusted life years between 1990 and 2019, depressive disorders are among the top ten conditions with the greatest rise in the burden of disease over the past few decades [3].

Depression in older adults is considered a geriatric syndrome, which is a complex set of multisystem impairments interrelated with multiple biological and psychosocial factors [4, 5]. Depression is associated with several chronic diseases, such as coronary heart disease, hypertension, diabetes mellitus, rheumatoid arthritis, and Alzheimer’s disease [6–8]. Older people with depressive symptoms are more likely to suffer from functional disability and dependence in activities of daily living than nondepressed individuals [9, 10]. This can lead to a decline in quality of life and an increase in the rates of falls and mortality [11–13].

Additionally, late-life depression is reciprocally associated with other geriatric syndromes, such as frailty and sarcopenia [14, 15]. Low muscular strength and slow walking are two of the key features of these syndromes [16]. Handgrip strength (HGS), as measured by a hand dynamometer, is a reliable indicator of overall muscle strength and a marker of aging and health status [17]. Gait speed (GS), as measured by timed tests for usual or maximum pace, represents physical performance and is a health indicator in older adults [18]. HGS and GS are of prognostic value for predicting age-related health problems, including cognitive dysfunction, falls, hospitalization, and mortality [17, 18]. Moreover, depressive disorders are probably associated with physical impairments, reflected in low HGS and GS [19, 20].

Previous studies have used various assessment tools for depression, categorized the physical function indices into different numbers of levels with varying cutoff points, and studied populations with dissimilar backgrounds. Although pooled estimations support the link between depression and HGS or GS, heterogeneity in studies should deter us from generalization to every population [19–21]. For instance, a study of six low- and middle-income countries showed that the strength of the association between HGS and depression was different among countries. The largest effect sizes were observed in Ghana and India, while the results were ambivalent in Mexico, Russia, China, and South Africa [22].

Depression has an estimated prevalence of 52% in Iranian older adults, which is higher than the worldwide reports [2, 23]. This difference can be attributed to the role of socioeconomic, cultural, and genetic determinants of depression that vary widely in different countries [24, 25]. Moreover, the physical function of Iranian people can be distinct; for example, the normative ranges of HGS in Iranian population were shown to be lower than the North American, West European, and Australian populations, while higher than East Asian populations. The decline in HGS by age is also steeper in Iranian older people than most of other countries [26]. To the best of our knowledge, no study has previously explored the association of HGS and GS with depressive symptoms in Iran. To understand this topic in the Iranian elderly population, we examined a sample of older adults residing in Birjand.

## Methods

### Study design and population

This is a cross-sectional study analyzing data from the first wave of the Birjand Longitudinal Aging Study (BLAS), which is a prospective cohort of community-dwelling older adults (≥ 60 years). The BLAS was approved by the research ethical committee of Endocrinology and Metabolism Research Institute of Tehran University of Medical Sciences (Approval ID: IR.TUMS.EMRI.REC.1396.00158) and ethical committee of Birjand University of Medical Sciences (Approval ID: IR.BUMS.REC.1397.282). The data for the first wave of the BLAS, including 1420 participants, were collected from October 2018 to April 2019. Eight trained researchers, consisting of nurses and physicians, each collected one of eight domains of data from the participants and their informants. The researchers that collected each domain of data were blind to other domains; for instance, those who evaluated HGS and GS were not aware of the depressive symptoms. A detailed elaboration on the study methodology has been published previously [27].

### Depressive symptoms

The nine-item Patient Health Questionnaire (PHQ-9) was used to assess depressive symptoms. We defined clinically significant depressive symptoms as scores ≥ 10, which is 88% sensitive and 88% specific for detecting MDD. The severity of depressive symptoms was categorized into minimal or none (0–4), mild (5–9), moderate (10–14), moderately severe (15–19), and severe (20–27) [28]. The reliability and validity of the Persian version of PHQ-9, employed in the present study, have been established in Iranian samples [29–31].

### Physical parameters

A digital hand dynamometer (Saehan Corporation, Changwon-si, Gyeongsangnam-do, South Korea), which was found to be reliable [32], was used to measure HGS in kilograms (kg), while the participant was sitting with the elbow bent at 90°. The measurements were repeated six times, three times for each of left and right hands, at least two minutes apart, and the largest value of the six attempts was used in analyses. We classified HGS into quartiles for each sex. Low HGS was defined in accordance with the definition of weakness in the Fried frailty phenotype as the lowest 20% of the cohort, adjusted for sex and body mass index (BMI) [33].

To measure GS, a researcher asked the participants to walk a 15-foot (≈ 4.57-meter) distance, preceded and followed by a 1-meter acceleration and 1-meter deceleration distances, at their usual pace three times. The maximum GS in meters per second (m/s) was calculated by dividing 4.57 by the shortest time in seconds, categorized into sex-specific quartiles, was used in analyses. Low GS was defined as the lowest 20% of the population, adjusted for sex and height, based on the definition of slowness in the Fried frailty phenotype [33].

### Covariates

Sociodemographic and other health-related data, including age, sex, marital status, education, occupation, income, chronic diseases, medications, and current smoking status were self- or informant-reported. Polypharmacy was defined as concurrent routine consumption of five or more medications [34]. To create the wealth index (WI), principal component analysis (PCA) was used to summarize ten variables that distinguished between relatively rich and relatively poor households (the ownership status of a refrigerator, vacuum cleaner, microwave oven, washing machine, dishwasher, water purifier, LED television, smartphone, automobile, and house). The PCA was run with ten binary variables, and the first component was considered the WI, which was then ranked into quintiles [35].

Height and weight were measured using a stadiometer (Seca GmbH & Co. KG., Hamburg, Germany) and a calibrated digital scale (Seca GmbH & Co. KG., Hamburg, Germany), respectively. BMI was calculated by dividing weight (in kg) by the square of height (in meters) and categorized into underweight (< 18.5 kg/m^2^), ideal weight (18.5–24.9 kg/m^2^), overweight (25–29.9 kg/m^2^), and obesity (≥ 30 kg/m^2^) [36]. Waist circumference was gauged with a nonelastic tape held horizontally just above the iliac crest in a standing position after a normal exhalation.

Physical activity was evaluated by the Longitudinal Ageing Study Amsterdam Physical Activity Questionnaire (LAPAQ), comprising 18 questions about the frequency and duration of six domains of activities over the last two weeks. The data were converted into the metabolic equivalent of task (MET) per day based on the intensity of each activity [37]. The Mini Nutritional Assessment (MNA) tool was used to rate participants’ nutritional status and divide them into normal nutritional status (≥ 24), at risk of malnutrition (17–23.5), and malnutrition (< 17) categories [38]. Cognitive function was evaluated by the six-item Cognitive Impairment Test (6-CIT), which is valid and reliable tool to assess temporal orientation, short-term memory, and attention [39]. The participants were categorized into normal cognitive function (6-CIT score ≥ 8) and impaired cognitive function (6-CIT score < 8).

### Statistical analysis

We described continuous and categorical variables as the mean ± standard deviation (SD) and frequency (count, percentage), respectively. We compared continuous and categorical variables in depressed versus nondepressed participants by independent samples t-test and Pearson’s chi-squared test, respectively. We performed univariable and multivariable logistic regression to examine the association of HGS and GS with the presence of clinically significant depressive symptoms and reported the results as odds ratio (OR) with 95% confidence interval (CI). To assess the association of HGS and GS with the severity of depressive symptoms, we conducted multiple multinomial logistic regression analyses and reported the results as relative risk ratio (RRR) with 95% CI.

We primarily selected the covariates included in the multivariable regression models (age, sex, marital status, occupation, WI, BMI, waist circumference, diabetes mellitus, hypertension, osteoarthritis, polypharmacy, nutritional status, current smoking status, physical activity, and cognitive function) based on previous studies [21]. We included all the mentioned covariates in the regression models that we called “full models” and reported in the Supplementary Tables 2 and 3 [see Additional file 1]. Moreover, backward stepwise selection was used to keep the most relevant variables (p value < 0.1) in the final regression models reported in Tables 1, 2, 3 and 4.

**Table 1:** Description of baseline characteristics and comparison of participants with and without clinically significant depressive symptoms

Characteristic		Total (*N* = 1348)	Depressive symptoms (*N* = 268, 19.94%)	No depressive symptoms (*N* = 1076, 80.06%)	***p***-value ^a^
Age, years (mean ± SD)		69.73 ± 7.53	70.66 ± 8.01	69.52 ± 7.39	0.027
Age groups (N, %)	60–69 years	753, 56.03	136, 50.75	617, 57.34	0.055
	70 − 79 years	422, 31.40	88, 32.84	334, 31.04	
	≥ 80 years	169, 12.57	44, 16.42	125, 11.62	
Sex (N, %)	Female	697, 51.71	191, 71.27	505, 46.93	< 0.001
	Male	651, 48.29	77, 28.73	571, 53.07	
Education (N, %)	Illiterate	605, 45.01	170, 63.43	435, 40.43	< 0.001
	Primary school	426, 31.70	66, 24.62	360, 33.46	
	High school	74, 5.51	8, 2.98	66, 6.13	
	Diploma	133, 9.90	14, 5.22	119, 11.06	
	Academic	106, 7.89	10, 3.73	96, 8.92	
Marital status (N, %)	Single	245, 18.24	72, 26.87	173, 16.09	< 0.001
	Married	1098, 81.76	196, 73.13	902, 83.91	
Occupation (N, %)	Retired	501, 37.28	45, 16.79	456, 42.38	< 0.001
	Employed	170, 12.65	28, 10.45	142, 13.20	
	Housekeeper	606, 45.09	179, 66.79	427, 39.68	
	Unemployed	67, 4.99	16, 5.97	51, 4.74	
Wealth index (mean ± SD)		0.002 ± 0.887	-0.312 ± 0.863	0.080 ± 0.875	< 0.0001
Current smoking status (N, %)	Non-smoker	1228, 91.37	240, 89.55	988, 91.82	0.237
	Smoker	116, 8.63	28, 10.45	88, 8.18	
Diabetes mellitus (N, %)	No	980, 72.92	190, 70.90	790, 73.42	0.405
	Yes	364, 27.08	78, 29.10	286, 26.58	
Hypertension (N, %)	No	585, 43.53	124, 46.27	461, 42.84	0.312
	Yes	759, 56.47	144, 53.73	615, 57.16	
Osteoarthritis (N, %)	No	1086, 81.17	195, 73.03	891, 83.19	< 0.0001
	Yes	252, 18.83	72, 26.97	180, 16.81	
Polypharmacy (N, %)	No	1137, 84.60	213, 79.48	924, 85.87	0.009
	Yes	207, 15.40	55, 20.52	152, 14.13	
Body mass index, kg/m^2^ (mean ± SD)		26.42 ± 5.32	26.33 ± 5.73	26.44 ± 5.22	0.757
Body mass index categories (N, %)	Underweight	63, 4.69	17, 6.34	46, 4.28	0.186
	Ideal weight	484, 36.01	104, 38.81	380, 35.32	
	Overweight	512, 38.10	89, 33.21	423, 39.31	
	Obese	285, 21.21	58, 21.64	227, 21.10	
Waist circumference, cm (mean ± SD)		95.24 ± 11.83	95.08 ± 12.30	95.28 ± 11.72	0.801
Physical activity, METs/day (mean ± SD)		4.03 ± 6.37	2.45 ± 4.52	4.42 ± 6.70	< 0.0001
Nutritional status (N, %)	Normal nutritional status	986, 73.36	144, 53.73	842, 78.25	< 0.0001
	At risk of malnutrition	343, 25.52	114, 42.54	229, 21.28	
	Malnutrition	15, 1.12	10, 3.73	5, 0.46	
Cognitive function (N, %)	Normal	574, 42.71	60, 22.39	514, 47.77	< 0.001
	Impaired	770, 57.29	208, 77.61	562, 52.23	
Handgrip strength, kg (mean ± SD)	Female	16.26 ± 5.96	15.76 ± 6.21	16.44 ± 5.86	0.179
	Male	29.59 ± 8.91	26.39 ± 8.95	29.99 ± 8.82	0.001
Gait speed, m/s (mean ± SD)	Female	0.91 ± 0.36	0.86 ± 0.33	0.93 ± 0.36	0.032
	Male	1.31 ± 0.47	1.05 ± 0.47	1.34 ± 0.46	< 0.0001

^a^ Two samples t-test for continuous variables and Pearson’s chi-squared test for categorical variables

**Table 2:** Association of physical parameters with depressive symptoms estimated by logistic regression analysis

Variable	Categories	Odds ratio (95% confidence interval)		
		Model 1^a^	Model 2^b^	Model 3^c^
Handgrip strength	Quartile 1	1.00 (reference)	1.00 (reference)	1.00 (reference)
	Quartile 2	0.49 (0.33–0.72)	0.53 (0.35–0 0.79)	0.61 (0.40–0.93)
	Quartile 3	0.54 (0.38–0.77)	0.56 (0.38–0.83)	0.68 (0.45–1.00)
	Quartile 4	0.53 (0.37–0.77)	0.56 (0.38–0.83)	0.80 (0.53–1.20)
Gait speed	Quartile 1	1.00 (reference)	1.00 (reference)	1.00 (reference)
	Quartile 2	0.79 (0.55–1.13)	0.83 (0.56–1.21)	0.93 (0.63–1.39)
	Quartile 3	0.65 (0.44–0.97)	0.60 (0.39–0.93)	0.81 (0.51–1.27)
	Quartile 4	0.39 (0.27–0.56)	0.45 (0.30–0.69)	0.66 (0.42–1.03)

^a^Univariable analysis

^b^Adjusted for age and sex

^c^For handgrip strength: adjusted for sex, wealth index, current smoking status, physical activity, polypharmacy, nutritional status, cognitive function, and osteoarthritis; for gait speed: adjusted for age, sex, wealth index, current smoking status, physical activity, diabetes mellitus, polypharmacy, nutritional status, cognitive function, and osteoarthritis

**Table 3:** Association of physical parameters with severity of depressive symptoms estimated by multiple multinomial logistic regression

Variable	Categories	Severity of depressive symptoms, Relative risk ratio^a^ (95% confidence interval)			
		Mild	Moderate	Moderately severe	Severe
Handgrip strength^b^	Quartile 1	1.00 (reference)	1.00 (reference)	1.00 (reference)	1.00 (reference)
	Quartile 2	0.76 (0.51–1.13)	0.47 (0.27–0.79)	0.60 (0.28–1.30)	1.20 (0.35–4.10)
	Quartile 3	0.72 (0.49–1.06)	0.55 (0.34–0.90)	0.70 (0.35–1.41)	0.51 (0.12–2.23)
	Quartile 4	0.76 (0.51–1.13)	0.67 (0.40–1.10)	0.65 (0.30–1.42)	1.47 (0.44–4.98)
Gait speed^c^	Quartile 1	1.00 (reference)	1.00 (reference)	1.00 (reference)	1.00 (reference)
	Quartile 2	0.76 (0.50–1.14)	0.92 (0.56–1.52)	0.69 (0.34–1.42)	0.61 (0.20–1.87)
	Quartile 3	1.36 (0.88–2.11)	1.03 (0.58–1.85)	0.89 (0.40–2.01)	0.51 (0.13–2.00)
	Quartile 4	0.72 (0.48–1.09)	0.77 (0.45–1.32)	0.39 (0.17–0.90)	0.14 (0.02–0.75)

^a^Relative to no or minimal depressive symptoms

^b^Adjusted for sex, wealth index, current smoking status, physical activity, polypharmacy, nutritional status, cognitive function, and osteoarthritis

^c^Adjusted for age, sex, wealth index, current smoking status, physical activity, polypharmacy, nutritional status, cognitive function, diabetes mellitus, and osteoarthritis

**Table 4:** Association of physical parameters with depressive symptoms estimated by multiple logistic regression, stratified by sex

Variable	Categories	Odds ratio (95% confidence interval)	
		Female	Male
Handgrip strength^a^	Quartile 1	1.00 (reference)	1.00 (reference)
	Quartile 2	0.81 (0.47–1.39)	0.43 (0.20–0.91)
	Quartile 3	0.85 (0.53–1.39)	0.56 (0.26–1.21)
	Quartile 4	0.98 (0.59–1.62)	0.78 (0.34–1.78)
	Low vs. normal	1.04 (0.71–1.54)	1.08 (0.62–1.89)
Gait speed^b^	Quartile 1	1.00 (reference)	1.00 (reference)
	Quartile 2	1.34 (0.81–2.22)	0.48 (0.24–0.96)
	Quartile 3	1.14 (0.66–1.96)	0.38 (0.15–1.00)
	Quartile 4	1.06 (0.61–1.87)	0.25 (0.11–0.57)
	Low vs. normal	0.86 (0.54–1.36)	1.75 (0.74–4.13)

^a^Adjusted for wealth index, current smoking status, physical activity, polypharmacy, nutritional status, cognitive function, and osteoarthritis

^b^Adjusted for age, wealth index, current smoking status, physical activity, polypharmacy, nutritional status, cognitive function, diabetes mellitus, and osteoarthritis

**Table 5:** Association of physical parameters with severity of depressive symptoms estimated by multiple multinomial logistic regression

Variable	Severity of depressive symptoms, Relative risk ratio^a^ (95% confidence interval)			
	Mild	Moderate	Moderately severe	Severe
Handgrip strength^b^	1.03 (0.77–1.37)	0.95 (0.65–1.39)	1.70 (0.90–3.21)	0.89 (0.34–2.35)
Gait speed^c^	0.81 (0.53–1.24)	0.88 (0.54–1.46)	1.12 (0.56–2.27)	0.42 (0.10–1.79)

^a^Relative to no or minimal depressive symptoms

^b^Adjusted for sex, wealth index, current smoking status, physical activity, polypharmacy, nutritional status, cognitive function, and osteoarthritis

^c^Adjusted for age, sex, wealth index, current smoking status, physical activity, polypharmacy, nutritional status, cognitive function, diabetes mellitus, and osteoarthritis

We performed a subgroup analysis to explore the association of HGS and GS with depressive symptoms in male and female participants. We also conducted a sensitivity analysis using HGS and GS as dichotomous variables to evaluate their association with depressive symptoms. All statistical analyses were conducted using Stata Statistical Software: release 16 (StataCorp LP, College Station, Texas, USA).

## Results

A total of 1420 individuals were included in the first phase of the BLAS. After excluding subjects with incomplete data, 1348 participants (mean age = 69.73 ± 7.53 years, 51.71% female) were analyzed in the present study. The average HGS was 16.26 ± 5.96 kg in women and 29.59 ± 8.91 kg in men. The average GS was 0.91 ± 0.36 in women and 1.31 ± 0.47 in men. The mean ± SD of the first through fourth quartiles of HGS and GS are reported in Supplementary Table 1 [see Additional file 1]. Clinically significant depressive symptoms (PHQ-9 scores ≥ 10) were observed in 268 (19.94%) individuals. Seven-hundred and nine (52.75%) individuals had no depressive symptoms, while 367 (27.31%), 179 (13.32%), 67 (4.99%), and 22 (1.64%) had mild, moderate, moderately severe, and severe depressive symptoms, respectively.

The sociodemographic and anthropometric characteristics of the participants are presented in Table 5. A larger proportion of participants with clinically significant depressive symptoms were female (71.27% vs. 46.93%, *p* < 0.001), illiterate (63.43% vs. 40.43%, *p* < 0.001), single (26.87% vs. 16.09%, *p* < 0.001), housekeeper (66.79% vs. 39.68%, *p* < 0.001), unemployed (5.97% vs. 4.74%, *p* < 0.001), cognitively impaired (77.61% vs. 52.23%, *p* < 0.001), suffering from osteoarthritis (26.97% vs. 16.81%, *p* < 0.0001), consuming multiple medications (20.52% vs. 14.13%, *p* = 0.009), malnourished (3.73% vs. 0.46%, *p* < 0001), and at risk of malnutrition (42.54% vs. 21.28%, *p* < 0001); compared to those without symptoms. They were, on average, less wealthy (WI: -0.312 ± 0.863 vs. 0.080 ± 0.875, *p* < 0.0001) and less physically active (METs/day: 2.45 ± 4.52 vs. 4.42 ± 6.70, *p* < 0.0001) than non-depressed participants. The means of GS were lower in both men (1.05 ± 0.47 vs. 1.34 ± 0.46 m/s, *p* < 0.0001) and women (0.86 ± 0.33 vs. 0.93 ± 0.36 m/s, *p* = 0.032) with depressive symptoms. The means of HGS were lower in men (26.39 ± 8.95 vs. 29.99 ± 8.82 kg, *p* = 0.001) and women (15.76 ± 6.21 vs. 16.44 ± 5.86 kg, *p* = 0.179) with depressive symptoms, but the mean difference in women was not statistically significant.

Table 1 summarizes the results of logistic regression analyses indicating the association of HGS and GS with the presence of clinically significant depressive symptoms. The participants in the second quartile of HGS and the fourth quartile of GS had the lowest odds of suffering from depressive symptoms. After controlling for potential confounders, individuals in the second quartile of HGS had 39% lower odds of having depressive symptoms compared to those in the first quartile (OR = 0.61, 95% CI: 0.40–0.93). The adjusted odds of having depressive symptoms in participants in the fourth quartile of GS was 34% lower than those in the first quartile; although this difference was not statistically significant (OR = 0.66, 95% CI: 0.42–1.03).

The relationship between the severity of depressive symptoms and the physical parameters estimated by multinomial logistic regression is demonstrated in Table 2. A higher HGS, especially the second vs. first quartile, was preventive against a moderate level of depressive symptoms, while a higher GS was more strongly associated with lower risks of moderately severe and severe symptoms. Participants in the second quartile of HGS were 53% less likely than those in the first quartile to suffer from moderate symptoms relative to minimal or no symptoms (RRR = 0.47, 95% CI: 0.27–0.79). The relative risks of having moderately severe and severe symptoms vs. minimal or low symptoms for participants in the fourth quartile of GS were, respectively, 61% and 86% lower than those in the first quartile (RRR = 0.39, 95% CI: 0.17–0.90; RRR = 0.14, 95% CI: 0.02–0.75; respectively).

Analyses stratified by sex showed that the association of HGS and GS with depressive symptoms was more prominent in the male population. While the odds of depressive symptoms in men in the second quartile of HGS was 57% lower than the first quartile (OR = 0.43, 95% CI: 0.20–0.91), and the odds of depressive symptoms in men in the fourth quartile of GS was one-fourth of those in the first quartile (OR = 0.25, 95% CI: 0.11–0.57); the results for women were ambivalent (Table 3).

When defined as dichotomous variables, HGS and GS were not significantly associated with the presence of depressive symptoms (OR = 1.07, 95% CI: 0.78–1.47; OR = 1.00, 95% CI: 0.67–1.49; respectively) after adjustment for potential confounding variables. Stratification by sex did not yield significant results in either subgroup (Table 3). Additionally, as demonstrated in Table 4, dichotomous HGS and GS were not associated with any of the severity levels of depressive symptoms.

## Discussion

The findings from this study provide valuable insights into the relationship between physical parameters and depressive symptoms in the Iranian elderly population. This cross-sectional study of community-dwelling older adults suggests that weaker handgrip and slower gait are associated with the presence and severity of depressive disorders, although we could not show a statistically significant association between GS and depression in our study. It is also worth noting that this study did not find a significant association when defining HGS and GS as dichotomous variables based on the Fried frailty phenotype [33]. This result highlights the importance of considering the nuances of physical performance and muscle strength rather than relying on binary categorizations, especially in the absence of specific cutoff points for Iranian populations.

Our findings are comparable to the existing literature on the association between physical impairments and depressive disorders. Several cross-sectional community-based studies of older adults conducted worldwide have demonstrated an association between HGS and depressive symptoms, as assessed by various tools, such as the PHQ-9, Geriatric Depression Scale, and Center for Epidemiologic Studies Depression scale [22, 40–44]. One community-based study also investigated the association between GS and depression, which yielded significant results [43]. In addition, longitudinal studies of community-dwelling older people demonstrated bidirectional inverse relationships between depressive symptoms and both HGS and GS. In other words, not only weakness and slowness lead to a higher risk of developing depressive symptoms, but depression also decreases the muscular strength and walking speed [45, 46].

Subgroup analyses in previous studies generally did not reveal heterogeneity among categories divided by sex, age, BMI, or physical activity [40, 41]. However, in our study, the association between depressive symptoms and physical indices was more significant in men. This sex variation was also reported by Zhang et al., who observed a more significant association between HGS and depressive symptoms in male cancer survivors [47]. An Indian community-based study of older adults also found that the association of HGS and depression was more prominent in men [40]. Conversely, a Korean study involving outpatients of a geriatric clinic found an association among GS, HGS, and depression only in women [48]. These differences may indicate variations in the physiological and psychosocial factors underlying the relationship between physical parameters and depression in different populations.

Interestingly, the odds of depressive symptoms were lower in the second quartile of HGS than in the other quartiles, suggesting a nonlinear relationship between muscle strength and depression. Likewise, Zhang et al. observed a similar cross-sectional pattern of nonlinear relationship in hospitalized Chinese older adults such that for HGS under 35.6 kg, higher strength was associated with lower risk of depression, while for HGS above 35.6 kg, the relationship lost significance [49]. Moreover, a longitudinal population-based study in 24 European countries showed a significant decline in depression risk with HGS increase only up to the level of 40 kg in men and 27 kg in women [50]. These findings, as well as ours, can hint at a potential protective effect of moderate, rather than high, HGS against depression. This pattern can be explained by nonlinear association of HGS and systemic immune-inflammatory index (SII), which is an indicator of overall level of immune activity and inflammation and is also associated with depression risk [51, 52]. Wu et al. showed that SII decreases with HGS increase when the level of HGS is under 2.48 kg per 1 kg/m^2^ BMI and SII plateaus when HGS is higher [51]. This might be linked to the anti-inflammatory and immunoregulatory role of interleukin-6 (IL-6) when excreted modestly from muscles during moderate exercise. However, long-term severe exercise makes muscles release higher amounts of IL-6, leading to its pro-inflammatory effects [53, 54].

Our results underscore the importance of evaluating HGS and GS in older adults and suggest the implementation of interventions aimed at improving physical fitness to enhance mood and reduce the risk of depressive disorders. Fortunately, HGS and GS can be modified by interventions such as strength training, resistance exercises, and nutritional supplements including whey protein, amino acids, and vitamin D [55]. Considering the bidirectional nature of this relationship, it is crucial to monitor depressive symptoms in people with sarcopenia and frailty and address their mood disorders to enhance their physical capabilities.

Although the exact mechanism underlying the relationship between depressive mood and physical dysfunction is not fully understood, hypotheses propose that physical inactivity and poor nutrition contribute to both depression and muscular weakness [21]. Older adults with faster gait speed and higher mobility are more likely to engage in social activities, which can reduce the risk of depression [56, 57]. The association between muscular dysfunction and depression may be explained by age-related oxidative stress. Aged muscle cells produce higher levels of reactive oxygen species (ROS) due to mitochondrial dysfunction, and excess ROS disrupts muscular fiber activation and contraction [58]. On the other hand, elevated ROS in the brain can lead to depression by causing neuronal damage [59]. Furthermore, a genome-wide association study suggests the possible involvement of specific single-nucleotide polymorphisms and genes in the genetic correlation between depression and HGS [60]. Additionally, depression and muscular dysfunction may both result from telomere shortening during cellular aging [61, 62].

Causality cannot be demonstrated in our study for two main reasons. First, the cross-sectional design hinders our ability to establish the temporality of events. Second, depression and physical function are multidimensional health issues influenced by various physiological and psychosocial factors. Although we adjusted our statistical model for several relevant variables, there may be numerous other factors that were not taken into account in our models.

The sample in our study was carefully selected to represent the population of community-dwelling older adults, thereby allowing for virtual generalization of the findings to the broader older population. However, the results may not be applicable to individuals with extreme conditions, as those with severe motor and cognitive dysfunction were excluded from the BLAS. Furthermore, this study does not encompass older adults residing in nursing homes or those admitted to hospitals. This should be noted that although the Saehan digital hand dynamometer is a reliable tool to measure HGS [32], its validity has not been evaluated; therefore, our results should be cautiously compared to studies using different brands of dynamometers.

Given the high prevalence and significant consequences of depression, even small effect sizes within the confidence intervals of our results can be considered clinically meaningful. Clinical interpretation should take into account the specific setting, goals, and available resources. Further research should explore the clinical utility of HGS and GS in managing depressive disorders by proposing a new screening tool that incorporates objective measures of physical function and comparing its accuracy with existing tools. Additionally, future studies should investigate the effectiveness of interventions aimed at improving physical performance in the prevention and treatment of depressive disorders.

## Conclusions

In conclusion, our study suggests a relationship between depressive symptoms and physical dysfunction in older adults residing in Birjand. HGS and GS are inexpensive and accessible measures that clinicians can utilize to evaluate musculoskeletal performance, which may also imply mental health status. It is crucial to address both physical and psychological signs and symptoms to optimize healthcare for older adults.

### Electronic supplementary material

Below is the link to the electronic supplementary material.

Supplementary Material 1

## Data Availability

The data that support the findings of this study are available from the corresponding authors upon reasonable request.

## Abbreviations

HGS: handgrip strength
GS: gait speed
MDD: major depressive disorder
BLAS: Birjand Longitudinal Aging Study
PHQ-9: Patient Health Questionnaire
BMI: body mass index
WI: wealth index
PCA: principal component analysis
LAPAQ: Longitudinal Ageing Study Amsterdam Physical Activity Questionnaire
MET: metabolic equivalent of task
MNA: Mini Nutritional Assessment
6-CIT: Cognitive Impairment Test
SD: standard deviation
OR: odds ratio
RRR: relative risk ratio
CI: confidence interval
