# Supplementary material for: The association of depressive symptoms with handgrip strength and gait speed in community-dwelling older adults: data from the baseline phase of Birjand Longitudinal Aging Study
Source: BMC Geriatr. 2024 May 3;24:393. doi: 10.1186/s12877-024-04944-z (PMC11069203; doi:10.1186/s12877-024-04944-z)
Supplement: Supplementary file 1 — Supplementary Material 1 [file 12877_2024_4944_MOESM1_ESM.docx]

Supplementary Table 1. Description of handgrip strength and gait speed quartiles

| Variable | Category | Mean ± standard deviation | | |
| --- | --- | --- | --- | --- |
|  |  | Female | Male | Total |
| Handgrip strength (kg) | Quartile 1 | 8.88 ± 3.66 | 19.58 ± 3.50 | 14.15 ± 6.44 |
|  | Quartile 2 | 14.25 ± 0.78 | 26.34 ± 1.63 | 20.75 ± 6.17 |
|  | Quartile 3 | 17.46 ± 1.19 | 31.83 ± 1.69 | 24.18 ± 7.32 |
|  | Quartile 4 | 22.77 ± 4.27 | 40.81 ± 7.83 | 30.81 ± 10.86 |
| Gait speed (m/s) | Quartile 1 | 0.51 ± 0.12 | 0.73 ± 0.18 | 0.61 ± 0.19 |
|  | Quartile 2 | 0.77 ± 0.06 | 1.13 ± 0.08 | 0.94 ± 0.19 |
|  | Quartile 3 | 0.97 ± 0.06 | 1.39 ± 0.06 | 1.11 ± 0.21 |
|  | Quartile 4 | 1.36 ± 0.29 | 1.78 ± 0.30 | 1.60 ± 0.36 |

Supplementary Table 2. Association of physical parameters with depressive symptoms estimated by logistic regression analysis

| Variable | Category | Odds ratio (95% confidence interval) | | |
| --- | --- | --- | --- | --- |
|  |  | Female^a^ | Male^a^ | Total^b^ |
| Handgrip strength | Quartile 1 | 1.00 (reference) | 1.00 (reference) | 1.00 (reference) |
|  | Quartile 2 | 0.81 (0.47 – 1.39) | 0.38 (0.18 – 0.80) | 0.61 (0.40 – 0.94) |
|  | Quartile 3 | 0.84 (0.51 – 1.38) | 0.46 (0.21 – 1.01) | 0.68 (0.45 – 1.02) |
|  | Quartile 4 | 0.96 (0.58 – 1.60) | 0.60 (0.26 – 1.39) | 0.81 (0.53 – 1.25) |
|  | Low vs. normal | 1.05 (0.70 – 1.57) | 1.00 (0.54 – 1.84) | 1.07 (0.77 – 1.49) |
| Gait speed | Quartile 1 | 1.00 (reference) | 1.00 (reference) | 1.00 (reference) |
|  | Quartile 2 | 1.34 (0.81 – 2.23) | 0.47 (0.24 – 0.95) | 0.93 (0.62 – 1.39) |
|  | Quartile 3 | 1.12 (0.65 – 1.95) | 0.37 (0.14 – 0.98) | 0.79 (0.50 – 1.25) |
|  | Quartile 4 | 1.03 (0.58 – 1.81) | 0.25 (0.11 – 0.56) | 0.64 (0.41 – 1.01) |
|  | Low vs. normal | 0.86 (0.54 – 1.37) | 1.75 (0.73 – 4.18) | 1.01 (0.68 – 1.52) |

^a^Adjusted for age, marital status, occupation, WI, BMI, waist circumference, diabetes mellitus, hypertension, osteoarthritis, polypharmacy, nutritional status, current smoking status, physical activity, and cognitive function (full model)

^b^Adjusted for age, sex, marital status, occupation, WI, BMI, waist circumference, diabetes mellitus, hypertension, osteoarthritis, polypharmacy, nutritional status, current smoking status, physical activity, and cognitive function (full model)

Supplementary Table 3. Association of physical parameters with severity of depressive symptoms estimated by multiple multinomial logistic regression

| Variable | Categories | Severity of depressive symptoms, Relative risk ratio^a^ (95% confidence interval) | | | |
| --- | --- | --- | --- | --- | --- |
|  |  | Mild | Moderate | Moderately severe | Severe |
| Handgrip strength | Quartile 1 | 1.00 (reference) | 1.00 (reference) | 1.00 (reference) | 1.00 (reference) |
|  | Quartile 2 | 0.72 (0.48 – 1.08) | 0.46 (0.27 – 0.80) | 0.56 (0.25 – 1.23) | 1.29 (0.37 – 4.52) |
|  | Quartile 3 | 0.66 (0.44 – 0.98) | 0.54 (0.32 – 0.89) | 0.64 (0.31 – 1.33) | 0.44 (0.10 – 2.02) |
|  | Quartile 4 | 0.67 (0.44 – 1.02) | 0.66 (0.39 – 1.12) | 0.57 (0.25 – 1.28) | 1.32 (0.37 – 4.70) |
|  | Low vs. normal | 1.12 (0.83 – 1.51) | 0.96 (0.65 – 1.44) | 1.97 (1.02 – 3.81) | 0.94 (0.34 – 2.58) |
| Gait speed | Quartile 1 | 1.00 (reference) | 1.00 (reference) | 1.00 (reference) | 1.00 (reference) |
|  | Quartile 2 | 0.77 (0.51 – 1.16) | 0.93 (0.56 – 1.55) | 0.70 (0.34 –1.44) | 0.59 (0.19 – 1.84) |
|  | Quartile 3 | 1.38 (0.89 – 2.15) | 1.05 (0.58 – 1.88) | 0.88 (0.38 – 1.99) | 0.46 (0.11 – 1.83) |
|  | Quartile 4 | 0.72 (0.48 – 1.09) | 0.76 (0.44 – 1.32) | 0.37 (0.16 – 0.87) | 0.13 (0.02 – 0.74) |
|  | Low vs. normal | 0.80 (0.52 – 1.22) | 0.87 (0.52 – 1.44) | 1.14 (0.56 – 2.31) | 0.50 (0.12 – 2.07) |

^a^Relative to no or minimal depressive symptoms, Adjusted for age, sex, marital status, occupation, WI, BMI, waist circumference, diabetes mellitus, hypertension, osteoarthritis, polypharmacy, nutritional status, current smoking status, physical activity, and cognitive function (full model)
